# Bridging Implementation Science and Human-Centered Design: Developing Tailored Interventions for Healthier Eating in Restaurants

**DOI:** 10.1007/s43477-024-00133-7

**Published:** 2024-09-09

**Authors:** Melissa Fuster, Shelby Hipol, Terry TK Huang, Uriyoán Colón-Ramos, Cara Conaboy, Rosa Abreu, Lourdes Castro Mortillaro, Margaret A. Handley

**Affiliations:** 1 Department of Social, Behavioral, and Population Sciences, Tulane University School of Public Health and Tropical Medicine, New Orleans, LA USA; 2 Steinhardt School of Culture, Education, and Human Development, Department of Nutrition and Food Studies, New York University, New York, NY USA; 3 Center for Systems and Community Design and NYU-CUNY Prevention Research Center, Graduate School of Public Health and Health Policy, City University of New York, New York, NY USA; 4 Department of Global Health, Milken Institute of Public Health, George Washington University, Washington, USA; 5 Hospitality Management Department, New York City College of Technology, City University of New York, New York, USA; 6 Partnership for Research in Implementation Science for Equity (PRISE) Center, Department of Epidemiology and Biostatistics, School of Medicine, University of California, San Francisco, CA USA

**Keywords:** Restaurant, Nutrition, Human-centered design, Implementation science, Behavior change wheel, Hispanic/Latin communities

## Abstract

Restaurants are important institutions in the communities’ economy with the potential to promote healthier foods but have been under-engaged in public health nutrition efforts. In particular, independently owned, minority-serving and minority-owned restaurants, remain under-represented in nutrition promotion efforts despite disproportionate burdens of diet-related health outcomes among minority populations. Addressing this gap in engagement, we undertook a process of co-designing and implementing healthy eating-focused interventions in two Latin American restaurants in New York City, combining the Behavior Change Wheel intervention development framework with a Human-Centered Design approach. Restaurant owners and chefs were involved in the research synthesis and solution development processes, resulting in two tailored interventions. This paper describes this co-development process and offers reflections and lessons regarding: (1) implementation research in community settings, (2) the application of Human-Centered Design to promote the uptake of community-based interventions on food and health equity, and (3) the combined use of Human-Centered Design and Implementation science in these complex community settings.

Diet-related non-communicable diseases, such as heart disease and diabetes, are among the leading causes of death globally. In the United States, ethnic and racial minority populations, including Hispanics, are disproportionately affected by these conditions (Daviglus et al., 2014). The pressing nature of this problem requires new ways of promoting healthier eating habits, moving beyond individual-focused interventions to those that address the social and physical environments influencing food choices. Moreover, in line with recommendations from the World Health Organization, health promotion must include collaboration with stakeholders to focus on their needs and strengths, developing interventions via participatory, context-specific, multi-level, approaches (WHO, 2017).

Foods away from home are increasingly important and have been associated with poor dietary outcomes (Kityo & Park, 2022; Liu et al., 2020; Meza-Hernández et al., 2023; Polsky & Garriguet, 2021; Salleh et al., 2021; Wellard-Cole et al., 2022). These foods can be acquired through a variety of restaurants, yet, most research has focused on fast foods, with restaurants serving ethnic foods remaining an under-studied and under-engaged sector (Fuster et al., 2021).

Restaurants are complex settings that require solutions that align business goals, consumer demands, and community health, necessitating innovative and theory-driven approaches to promote change. Having restaurants facilitate healthier choices may seem at odds with the perceived lower profitability of healthier choices, related to whether owners view there is a demand for such offerings (Glanz et al., 2007). Moreover, restaurants serving ethnic or cultural foods face unique challenges, given customer expectations for authenticity in these cuisines, even when standards for authentic dishes are highly debatable and subjective depending on customer knowledge and perception of the given cuisine (Shahrin & Hussin, 2023). These aspects make Latin-American restaurants a unique and important sector for further engagement and research, with potential lessons learned for other restaurants serving ethnic cuisines in global contexts.

Our work sought to understand the complexity inherent to changing consumer nutrition food environments in Latin American restaurants, addressing contextual barriers in the design and implementation of interventions to improve intervention tailoring within diverse community contexts. This study applied human-centered design (HCD) approaches in tandem with theoretical frameworks from implementation science. We worked with two Latin American restaurants in New York City to develop tailored interventions with the goal of increasing the sales of healthier menu items. The intervention and implementation outcomes of the resulting interventions have been published elsewhere (Fuster, Dimond et al 2023a). In brief, the resulting interventions were rated as acceptable among restaurant owners, staff, and customers, and positive changes were sustained by the partner restaurants. However, these changes yielded modest increases in sales of healthier options in one restaurant and no significant changes in sales of healthier options in the second restaurant (Fuster, Dimond et al 2023a). To further understand the intervention development process that led to these outcomes, this manuscript describes the integrated application of HCD and the Behavior Change Wheel framework (Michie et al., 2011) to glean insights into how to engage independently owned restaurants, a difficult-to-reach and complex sector.

## Theoretical Framework and Approach: The Behavior Change Wheel and Human-Centered Design

We applied the Behavior Change Wheel, a comprehensive and systematic framework that synthesizes 19 frameworks of behavior change found in the literature (Michie et al., 2011), as the theoretical framework guiding the intervention design. The Behavior Change Wheel is guided by the Capability, Opportunity, and Motivation for Behavior (COM-B) Model, which approaches behavior change as the result of the interaction of its main components: Capability is addressed as physical (i.e., whether the individual has the needed skills to engage in the desired behavior, such as offering healthy foods on the menu) and psychological (i.e., an individual’s comprehension and knowledge, such as about healthy alternatives to population dishes). Opportunity encompasses the external factors influencing the behavior, including physical (i.e., aspects of the built and food environments that influence behavior) and social opportunity (i.e., cultural and social norms that influence the behavior, such as beliefs that restaurants are places to celebrate through rich foods). Lastly, motivation encompasses the internal processes that direct behavior, including reflective (i.e., evaluations and plans) and automatic (emotions) motivation (Michie et al., 2011). COM-B facilitates a better understanding of what needs to change for a specific behavior to happen and results in a behavioral diagnosis with related strategies, or intervention functions, that can be applied to facilitate change and the desired behavior, as part of the Behavior Change Wheel process. The Behavior Change Wheel was selected given its comprehensive and practical approach to promote behavior change, evidenced in its wide application, including in the promotion of healthier eating behaviors (Beck et al., 2019; Craveiro et al., 2021; Lucas et al., 2020; Timlin et al., 2021; Willmott et al., 2021).

We complemented the Behavior Change Wheel with HCD to directly help move insights into actionable innovations that restaurant owners and staff could realistically implement and sustain. HCD can be defined as the use of a designer’s mindset and tools to collaboratively develop solutions alongside the end users that are constructive, experimental and rooted in customer needs and contexts. For this project, that meant working alongside restaurant owners, chefs and staff to co-create potential healthy eating innovations that could appeal to their customer needs and wants. We selected this approach given its potential to create needed innovations for chronic disease prevention by acknowledging and empathizing with customer needs, context and preferences as well as the restaurants’ ability to implement and sustain these needed innovations. The approach applies abductive and reductive processes to create innovations through critical and creative thinking (Holeman & Kane, 2019; Huang et al., 2018; Matheson et al., 2015).

**Fig. 1:**
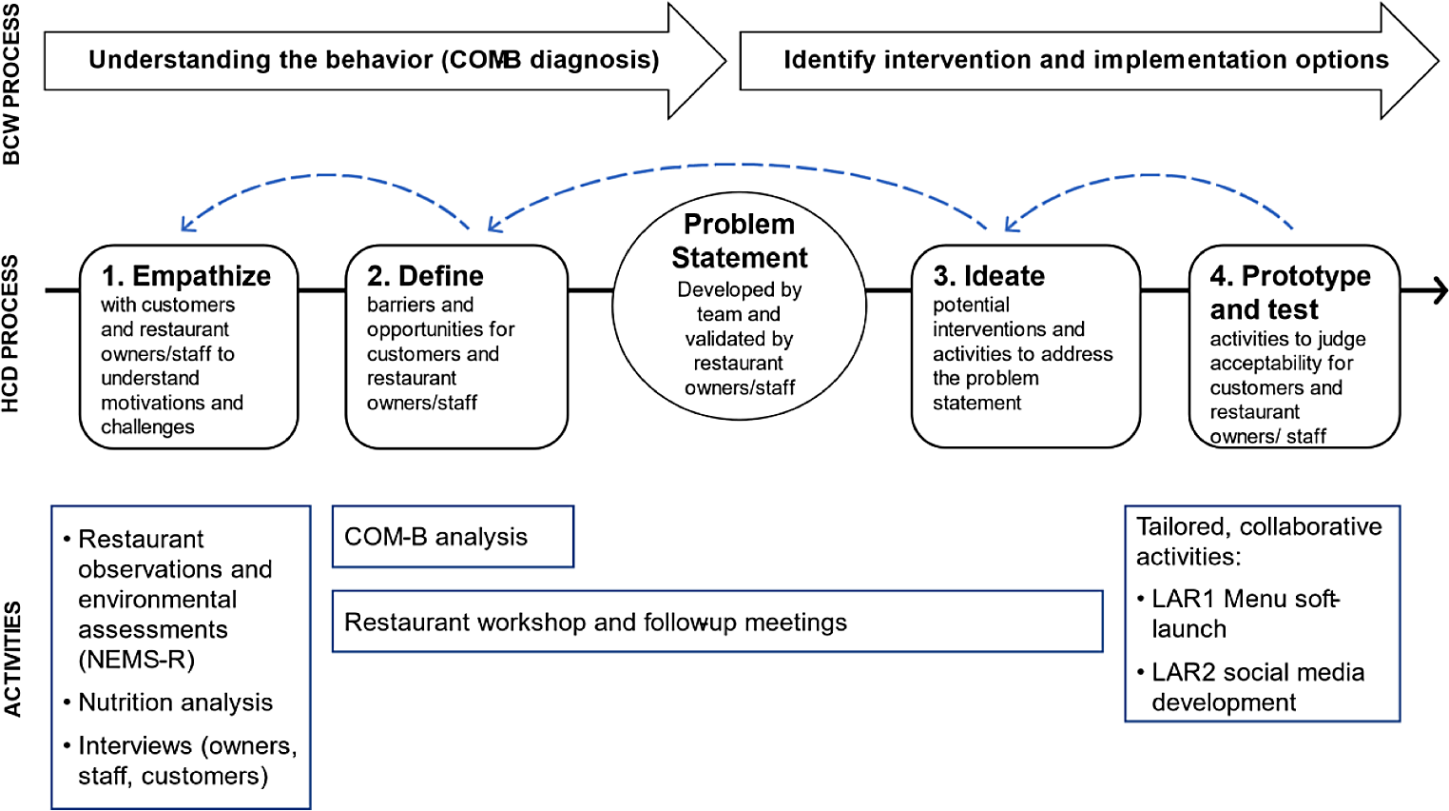
Study Overlapping Frameworks/Processes and Associated Activities. The Human-centered design (HCD) process was adapted from (Liu, 2016) and the Behavior change wheel (BCW) process adapted from (Michie et al., 2011). *Abbreviations:* COM-B: Capabilities, Opportunities, and Motivation for Behavior Model, NEMS-R: Nutrition Environment Measurement Survey for Restaurants, and LAR: Latin American Restaurant

In our work, we followed the processes outlined via the Behavior Change Wheel framework in parallel with those undertaken as part of HCD (Fig. 1). The first stage in the Behavior Change Wheel process is to understand the behavior — paralleling the empathize and define stages of HCD — to arrive at a problem statement. We then used iterative ideation and prototyping alongside the Behavior Change Wheel application of theory to identify intervention and implementation options (Fig. 1).

The process was undertaken by a multidisciplinary team to account for and include different perspectives and motivate transdisciplinary innovation. Our design team incorporated diverse perspectives beyond public health experts and practitioners, including nutrition and culinary experts, hospitality management, designers, and public health researchers. Using insights about consumer preferences from the formative research, described next, the design team collaborated with the end users – restaurant stakeholders – through formal and informal consultation activities to plan, validate and analyze potential innovations.

## Formative Research

The work presented in this manuscript builds on previous, formative research. We first conducted a scoping review of peer reviewed studies and industry reports examining healthy eating promoting changes in restaurants, including those motivated by public health interventions and those initiated by restaurants in the past twenty years. This work has been described in detail elsewhere (Fuster et al 2021). In brief, the review revealed differences in the type of strategies initiated by public health interventions compared with those initiated by restaurants. Changes in food availability (e.g., adding salads to menus, providing vegetarian options) were more common among corporate restaurants, while environmental facilitators (e.g., providing nutrition information, promoting healthier offerings) were more commonly initiated by public health researchers and associated with independently-owned restaurants. However, the scoping review revealed that most of these initiatives are taking place in corporate restaurants. The work also showed the need to apply practice-based evidence in the field and account for restaurant business models to tailor interventions and policies for sustained positive changes in these establishments (Fuster et al 2021).

Research was also undertaken to examine restaurant and customer perspectives concerning the healthy eating promoting strategies uncovered in our review. This included qualitative research with Latin-American restaurant owners and staff documenting their experiences, perspectives, barriers, and facilitators concerning healthy eating promoting strategies. This work showed that the most acceptable strategies for the Latin-American restaurant stakeholders that participated in the study were those that highlighted existing healthier items in the menu or via promotional efforts, followed by increasing healthy offerings. The least acceptable were the inclusion of nutrition information and reduced portions (including half-portions). This formative work also helped the academic team enumerate the resources needed for implementing the suggested changes, including nutrition knowledge, additional expertise (e.g., design, social media, culinary skills), and assistance with food suppliers and other restaurant operational logistics (Fuster et al 2022). The customer perspective included a survey with individuals that visit or order from Latin American restaurants on at least a monthly basis to examine perspectives and barriers concerning healthier eating in Latin American restaurants, applying the COM-B model. The examination revealed that while a greater proportion of the respondents had the capability to select healthier choices at Latin American restaurants, motivation and opportunity were relatively low (Fuster, Santos et al 2023b). This previous work informed the activities conducted at each partner restaurant, detailed below.

## Study Context and Restaurant Outreach

This project took place in New York City, where 28.9% of the population identifies as Latino/Hispanic (US Census, 2022). Our formative research began in February 2020. Restaurant outreach began in the Summer of 2020, initially adapted to a virtual approach given the onset of COVID-19. We used social media as an initial point of contact, as well as community networks and word of mouth. This initial effort included respondents representing a total of 13 restaurants, with 10 of those located in New York City, the location of the study team at the time. From this initial sample, five restaurants expressed interest in working with us in the development of tailored innovations. However, three of those dropped out due to operational issues. Specifically, one restaurant had to temporarily close due to issues with the kitchen equipment. Another restaurant was undergoing an internal dispute over ownership. The third restaurant was lost to follow-up due to the owner not being responsive when the team sought to schedule initial discussions to review the collaboration plan. These restaurants were all independently owned and relatively small in terms of size and staff capabilities (fewer than 10 employees), where the owners usually took on multiple roles (e.g., cooking, serving, managing). This was further compounded by the staff shortage that came as a result of COVID-19 (Sugar, 2022).

We collaborated with two Latin-American restaurants (LAR1 and LAR2). LAR1 was a counter-style restaurant located in a food hall, serving Puerto Rican food. Upon first contact, the restaurant had been in business for less than a year. As a counter-style restaurant, they were able to manage the initial COVID-19 closures because delivery and take-out were a core part of their business model. LAR2 was a full-service Mexican restaurant which had been open for two years prior to this project. They had to make major adaptations to the business upon the onset of COVID-19, including a limited delivery service and the temporary selling of packaged goods. While our engagement with the partner restaurants occurred during some COVID-19 restrictions, the intervention development process took place during the period of gradual opening when restaurants were allowed to open at full capacity starting in June 2021 (*A Demographic Snapshot*, 2020).

The two partner restaurants received a collaboration process overview document detailing the intervention development and testing activities, including the engagement and data needs, as part of the consent process for their participation in the process and pilot study. Incentives for participation included the provision of market research insights from our data collection efforts, the covering of intervention-related costs, stipend for staff and owners to participate in interview efforts (pre- and post-intervention testing period, $50/interview), and a $300 payment to the restaurant upon the completion of the activities.

## Intervention Development Process

Building from our formative research, the following sections present the intervention development process as detailed in Fig. 1. The activities specific to the partner restaurants began in June 2021, lasting a total of 4 months, concluding with the implementation of the tailored innovations. The engagement with both restaurants happened in parallel, beginning in June 2021 in LAR1 and in July 2021 in LAR2. We first describe the HCD process and then the Behavior Change Wheel process, occurring simultaneously (Fig. 1).

### Stage 1: Empathize (Discover)

The intervention design process began with an information-gathering stage that aimed to build understanding and empathy with the full scope of our end users, in this case, the restaurant owners, staff, and customers. This stage involved contextual structured observations, immersions, and interviews. Approaches and insights are presented below by first focusing on the context (the partner restaurants) and then examining the users within those contexts.

#### Contextual Insights: Environment and Menu Assessments

We gathered key insights from the partner restaurants using semi-structured interviews with owners and staff (completed during the formative research, see Fuster et al 2022), non-participant structured observations, an assessment of the consumer nutrition environment, and the nutritional analysis of best-seller items to discover potential areas for innovation or for enhancement, with the goal of motivating healthier choices.

The structured observations involved the systematic recording of phenomena in a given context, using a fly-on-the-wall technique where researchers watch activities as an unobtrusive observer to avoid changing people’s behaviors (Penin, 2017). We carried out onsite and virtual observation exercises. For onsite observations, we developed an observation protocol that incorporated the AEIOU of design: activities, environment, interactions, objects and users. Activities refers to what people do and how they do it (i.e., standing in line, reviewing menus, customizing orders and answering/asking questions). Environment refers to the restaurant *servicescape*, including the layout, size, atmosphere, and appearance, evaluated using all five senses. Interactions include people-people, people-object, and people-environment interfaces or exchanges. Objects can be understood as touchpoints, or the physical materials that support or facilitate service (i.e., serveware, signage, technology). In this exercise, Users included customers, staff and others (purveyors, neighboring restaurant personnel, and online customers) (Penin, 2017). Two trained research assistants visited the partner restaurants to carry out the observations, collecting and taking note of independent observations and then comparing and merging their fieldnotes and insights. During this observation exercise, the trained research assistants also applied the Nutrition Environment Measurement Survey for Restaurants (NEMS-R), a validated measure to examine environmental barriers and facilitators for healthier choices in restaurants, including an assessment of the menu and environmental factors (e.g., promotional materials, pricing) (Saelens et al., 2001). This was complemented by observations of the partner restaurant’s social media activity, focusing on Instagram as the main medium for restaurant promotion during COVID-19, as indicated by our restaurant partners. We reviewed daily posts for a period of one week, coding these according to the image content (i.e., food, alcohol, community, environment images), and, if the image presented foods, we coded these to note potentially healthy foods (i.e., showcasing vegetables, non-fried items). The inductive coding process was done by two trained research assistants and reconciled during team meetings. This was complemented by reviewing promotional material including website and others, to get a sense of the restaurant brand and how the cuisine was defined and promoted.

The environmental analysis revealed potential areas for improvement and emerging differences between the partner restaurants (Table 1). We found fewer facilitators and more barriers to healthier choices in LAR1, particularly the emphasis on large portions, a prominent display of fried snacks, and the low availability of healthier options. While LAR2 did not actively promote the existing healthier choices, the overall environment did not encourage over-eating. Contrary to LAR1, LAR2 did not encourage large portions nor had unhealthier options (Table 1). The menu provided the option for half-portions for some dishes. When comparing the foods available, almost none of the dishes in LAR2 were fried and the restaurant included a healthy main-dish salad, an item not present in LAR1. LAR1 did offer a potentially healthy side — a green salad — although, according to owner and chef interviews, this was not often purchased by customers.

**Table 1:** Selected Nutrition Environment Measurement Survey indicators by Partner Restaurant (LAR)

	LAR1	LAR2
***Food Availability***		
Whole grains	No	No
Fruit without added sugar	No	No
Nonfried, non-starchy vegetable side	Yes	Yes
Main dish salad	No	Yes
100% fruit juice	No	Yes
***Facilitators to Healthy Eating***		
Reduced/half portions of main dishes offered	No	Yes
Healthier options highlighted on site / menu	No	No
Smaller portions cost less than regular ones	NA	Yes
***Barriers to Healthy Eating***		
Large portions encouraged	Yes	No
Unhealthy options highlighted on site / menu	Yes	No
Healthier items cost more than comparable, regular items	No	No

We also conducted a nutrition analysis of the restaurants’ top-five sellers or dishes that best captured the essence of each restaurant, as identified by the partner restaurant owners and chefs. We used Nutrium, a nutrient analysis software based on the USDA 2018 nutrient database to calculate the nutrient profile for each recipe, including calories, fat, saturated fat, fiber, and sodium. The USDA Guidelines for Americans, 2020–2025 and the FDA Regulatory Requirements for Nutrient Content were then used to interpret the nutrient profiles from each recipe (*Dietary Guidelines for Americans:*^2020–2025^, ^2020^; FDA, 2013). To help contextualize the calorie count, the recommended daily allowance for calories published in the USDA Guidelines was divided by three, as a standard procedure to divide total daily allowances by the typical number of main meals consumed each day in the United States (*Dietary Guidelines for Americans:*^2020–2025^, ^2020^; FDA, 2013). While we recognize that there tend to be more than three eating episodes in a day, we felt apportioning calories in this manner was a realistic and consistent way of accounting for calories. Nutrient analyses were also compared to the FDA regulatory requirements for nutrient content claims (FDA, 2013). This provided a specific metric with which to compare total fat, saturated fat, sodium, and fiber.

Our analysis revealed the more popular menu items from both restaurants were high in both total fat and saturated fat (Table 2). LAR1 menu options were all high in sodium. When the results were shared with the chef, he attributed this to the use of salt-based seasoning blends. At the same time, as a result of the incorporation of beans and green plantains in their dishes, the restaurant had some items that provided at least 10% of the daily allowance of fiber. Conversely, all but one of LAR2 dishes met the criteria for healthy regarding sodium content and all provided at least 10% of the recommended daily allowance of fiber (Table 2). Hence, confirming the NEMS-R menu assessment, LAR2’s menu seemed to offer more healthy options for diners.

**Table 2:** Nutrition Analysis of the Top Five Sold Items in Partner Restaurants

Indicator (1/3 RDA)^a^	Indicator quantities, mean (min-max)		Count of top-5 dishes meeting recommendations	
	LAR1	LAR2	LAR1	LAR2
Calories (666–800)	552 (284–1186)	505 (243–841)	NA	NA
Fat (g) (7–12 g)	26 (1–91)	32 (12–76)	1	0
Saturated Fat (g) (< 3.3 g)	7 (0–24)	8 (2–28)	1	1
Sodium (mg) (< 766 mg)	1408 (664–2219)	330 (69–788)	0	4
Fiber (g) (9.3 g)^b^	3 (3–13)	7 (3–10)	2	5

^a^Daily Recommendation according to the 2020–2025 Dietary Guidelines for Americans: The range shown in parenthesis represents one-third of the Recommended Daily Allowance, adjusting for a single meal within a daily 3-meal pattern

^b^ Based on daily value (28 g per day) for adults, but recommendation varies by sex and age (FDA, 2013)

^c^ Based on the FDA Food Labeling Guide, dishes are denoted as healthy based on the following criteria: Total Fat: < 5 g fat/amount customarily consumed; Saturated Fat : <2 g sat fat / amount customarily consumed; Sodium: *≤* 600 mg / serving; Fiber: At least 10% of the daily value (FDA, 2013)

#### Insights from the Customer Experience and Perspectives

Customer perspectives were gathered via immersion, on-site short interviews, and contextual structured observations, building on insights gained from the formative research. Immersions refer to team members taking on the role of customers at each restaurant to understand the experience from the perspective of the customers rather than just as researchers. The team practiced immersion to best understand the customer experience by becoming customers, ordering from partner restaurants and others serving similar cuisines. The team took self-reflective notes on the process, documenting their experience approaching the menu, including which dishes called their attention and whether the menu promoted or facilitated healthful orders. The resulting notes were discussed in team meetings, providing insights on their experience as customers, further facilitating our building empathy with the end user. This was complemented with short, semi-structured customer interviews conducted during the structured context observation exercise described above. Two trained team members carried out the interviews with a convenience sample of potential customers in the vicinity of the restaurants (6 per restaurant). All interviews were completed on the same day for each location, during the work week. In LAR1, the interviews took place around lunchtime, and in LAR2 the interviews took place in the late afternoon, given the different opening hours in these locations. These were individuals who might not necessarily be visiting the restaurant but who provided insights about potential customers to get a sense of their needs and preferences, which might not be met by the restaurant in its current state. The short structured interviews captured general experience with Latin-American restaurants, previous experience with the partner Latin-American restaurant (i.e. Have you eaten at a Latin American restaurant? If yes, how was your experience? If no, what would motivate you to eat at [partner Latin American restaurant]? ), and opinions concerning healthier eating at Latin American restaurants including soliciting ideas about potential changes. We collected minimal demographic information (gender, age, race/ethnicity).

**Fig. 2:**
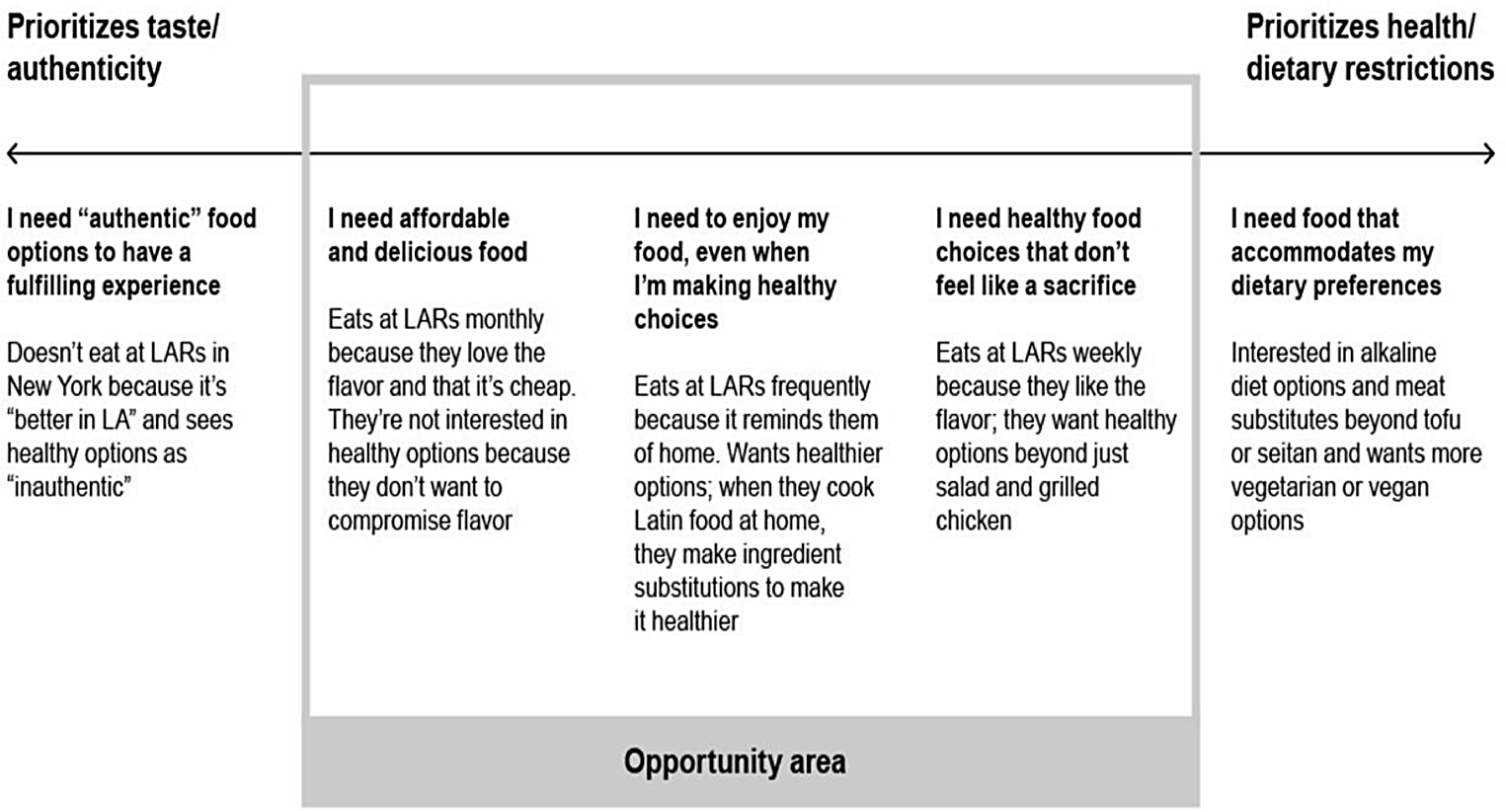
Consumer Priorities Spectrum and Identified Opportunity Area

The information gathered was synthesized using two approaches: The development of customer personas and the application of the COM-B Model. Personas are broad archetypes based on the data meant to portray potential end users for the team to keep in mind throughout the rest of the HCD process (Pearl & Intriligator, 2023). In this case, the personas were loose consumer archetypes based on interviewee’s stated preferences and priorities around healthy eating and their relationships and associations with Latin American restaurants, complemented by our additional data collection experiences. We reviewed the qualitative interview notes, distilling them down to each person’s most basic needs within them. Rather than defining every possible reason why people went to Latin American restaurants, we aimed to identify and map out some general themes so that we could tailor our interventions for the people who were most likely to benefit from or be interested in them. This resulted in five personas, showcased in Fig. 2, mapped on a spectrum from prioritizing taste/authenticity to prioritizing health/dietary preferences. We used these personas to understand not only what was important to customers but also to identify which customers might be receptive to healthier options at Latin American restaurants. In conversations with the restaurant owners, as part of the next phase in the process (to be expanded below), we used this spectrum to illustrate that there was an audience that wanted healthier options, but that we would not be able to please everyone, such as customers following strict or restrictive diets.

**Fig. 3:**
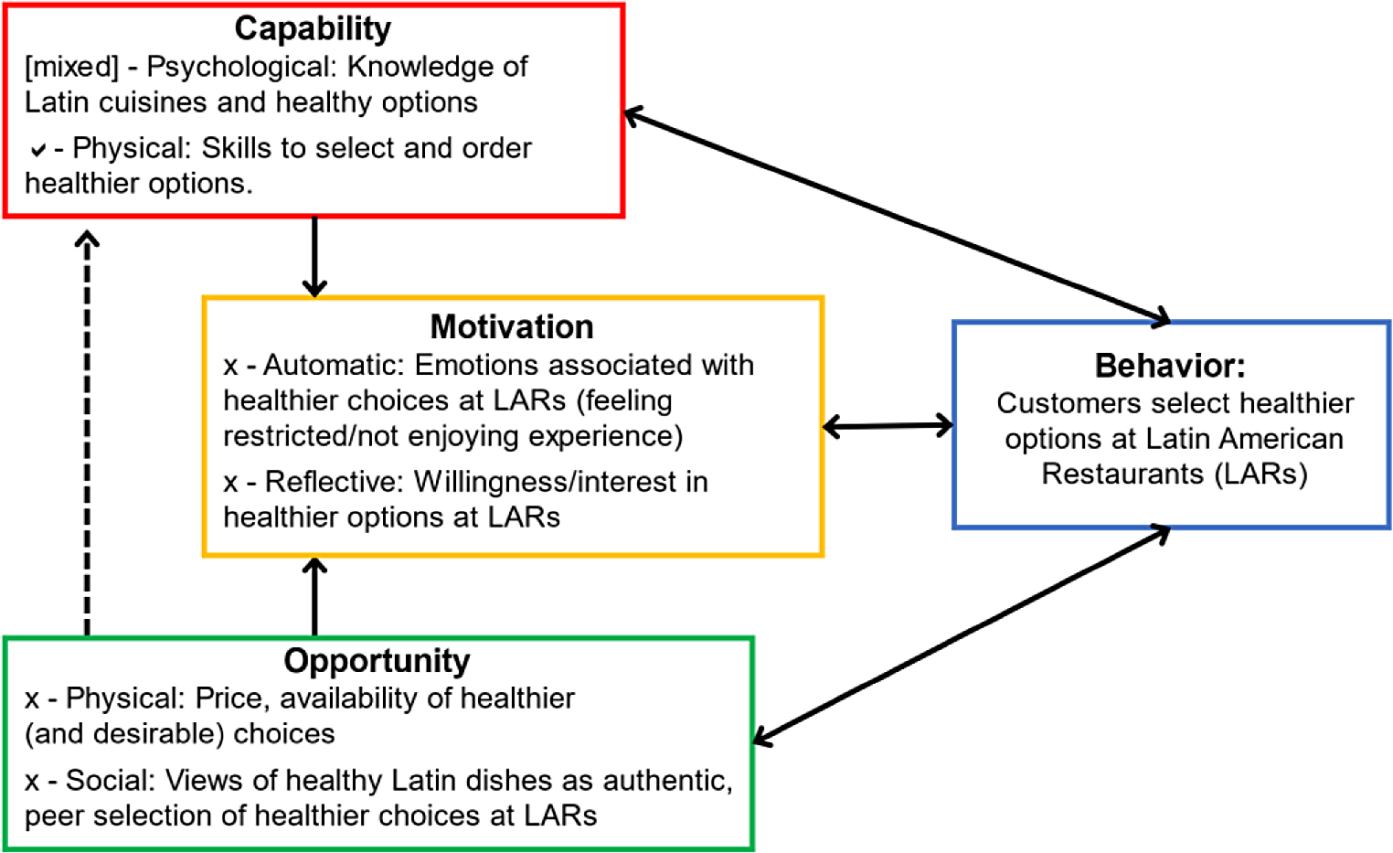
COM-B Model Examining Customer Healthier Food Choice. x denotes areas that are lacking (barriers),✓denotes facilitators, and [mixed] denotes mixed results. Model based on formative and Empathize stage research

Our formative work surveying Latin American restaurant customers provided initial insights into perceived barriers and facilitators for healthier choices in Latin American restaurants mapped using the COM-B Model (Fuster, Santos et al 2023b). We used our immersions and short interviews to develop the model further (Fig. 3). The COM-B diagnosis revealed the need to address psychological capability (knowledge), motivation (Automatic and reflective), and opportunity (physical and social) that would lead to ordering healthier choices at Latin American restaurants. Low knowledge concerning Latin American cuisines among the people interviewed led to the view of healthier options as not being authentic or an attempt to whitewash the cuisine. Specifically, there was a lack of knowledge concerning healthier ingredients (such as quinoa being native to South America) and cooking techniques. However, motivation and opportunity were more salient aspects. Motivation to select healthier options was thwarted by social opportunity (social and cultural norms) and physical opportunity (the availability of healthier options that are also perceived as enjoyable). In general, eating out was seen as a treat, where health was not the main focus. This was also expressed through the notion that “I can cook/eat healthier at home”.

### Stage 2: Define

The information gathered during the Empathize stage was used to define what needs to change, as shown in the COM-B analysis. This stage was undertaken with partner restaurants. We carried out workshops to share and discuss the results of our formative and discovery/empathize research and began brainstorming ideas for change (as part of the ideation process, presented in the next section). Each workshop lasted about three hours, engaging the owner and staff members (chef and marketing staff in LAR1 and chef in LAR2) through hands-on activities to facilitate the collaborative problem definition. The workshop was carried out onsite. We were able to discuss our findings within each restaurant context, which allowed us to deepen our understanding of the partner restaurants and validate the initial insights gathered as part of Stage 1. The discussion was focused on addressing opportunity and motivation for customers to engage in healthier choices – as the main areas of concerns revealed in the COM-B analysis (Fig. 3).

In LAR1, a key area for change was the need to increase the availability of healthy options – addressing physical opportunity through the environmental restructuring intervention function. While the establishment offered a side green salad, this was not selling, leading to food waste and consideration for elimination from the menu. This translated into the question: “How might we increase the availability of attractive healthy offerings, specifically, non-starchy/non-fried options?” with the related target behavior of having the chef increase the availability of attractive/innovative non-starchy/non-fried menu options. In LAR2, our analysis revealed that while the restaurant was already offering potentially healthy and innovative options, they faced the issue of some customers not accepting these offerings as authentic, pressuring the restaurant to offer less healthful options. This translated to “How might we change perceptions of healthy offerings as being inauthentic/not traditional?”, with the associated target behavior of having the restaurant owner increase social media messaging to promote healthier options as palatable and authentic. The emphasis on perceptions was geared to changing knowledge (psychological capability) and reflective motivation, COM-B drivers associated with the education intervention function.

### Stage 3: Ideation

Once we defined what needed to change via the tailored “How might we” questions for each restaurant, these were used to guide an ideation session as part of the onsite workshop described above (see Stage 2). The session was facilitated through an initial presentation of ideas developed by the team, with more ideas added through discussion with the restaurant partners. We then engaged the restaurant partners in a sorting activity, where the ideas were sorted in three piles: Yes, No, and Maybe. In this exercise, the sorting process incorporated elements of the Behavior Change Wheel APEASE criteria, which stands for Acceptability, Practicability, Effectiveness, Affordability, Spill-over effects, and Equity (Michie et al., 2011). Among these criteria, our sorting process revealed the importance of acceptability, practicability, and affordability, when discussing potential changes with our restaurant stakeholders. Evaluation of these elements was guided by perceived limitations around cost, space, equipment, and staff capabilities, further detailed below by partner restaurant.

In LAR1, the focus was under the Behavior Change Wheel environmental restructuring intervention function to facilitate healthier choices. When ideas were sorted, those grouped under “yes” were ideas that were perceived as the most acceptable, practicable, and affordable. Some ideas involved incorporating new vegetables and healthier ingredients, including using farro or other whole grains, traditional vegetables (e.g., cabbage, squash, taro) and different types of beans. Resulting from this, another change discussed was redesigning the menu to create a small selection of pre-built bowls, including new healthier combinations to facilitate customer choice. Ideas that did not rise to the top (classified as no and maybe) were those proposing to change portion sizes, cooking techniques (e.g., replacing frying), and incorporating plant-based alternatives to meat (e.g., tofu). The proposal to offer half-portions for bowls also failed to meet the affordability criteria, as the owner noted the need to purchase additional bowl sizes as an added cost. Changes in cooking technique and the provision of meat substitutes was deemed as unacceptable for the restaurant, given perceived customer expectations and the vision for the restaurant.

In LAR2, the ideas were focused on how to change perceptions of the cuisine and promote healthier dishes on the menu. The ideas that were seen as the most acceptable, practicable, and affordable were those under the Behavior Change Wheel education intervention function, as ideas related to social media messaging around offerings, highlighting ingredients in connection to the country of origin and its sensorial qualities. The owner expressed interest in this, given the potential to increase social media engagement as part of restaurant promotional efforts, as a key factor for augmenting customer outreach and sales. Beyond social media, a second idea was having servers promote healthier offerings when talking to customers, including the co-development of training resources, but this was not seen as practicable, given the owner and staff time constraints, compounded by the potential for ongoing staff turnover. Other ideas discussed were related to potential environmental restructuring to motivate healthier choices, such as eliminating less healthful offerings, adding more healthful offerings, and providing healthful markers in the menu to facilitate customers selecting these choices. However, while these were acceptable, the owner did not see these as affordable or practicable at the moment, given his time constraints and the lack of staff to delegate these changes on. The completion of the in-person workshop was followed up with a team debriefing meeting, where ideas were further discussed, including potential effectiveness, spill-over effects, and equity considerations, as part of the APEASE criteria.

### Stage 4: Prototyping and Resulting Interventions

The takeaways from the workshops were shared with the owners and staff as part of a subsequent meeting, where ideas were narrowed down for initial trials and quick tests (prototypes), prior to full implementation. During these meetings, we worked with the restaurants to devise a plan of action to test and refine the ideas. While we provided support, the processes were led by the restaurants, including when and how to test the ideas and their benchmark for success. This process took several meetings with the partner restaurants, allowing for the final intervention to be refined and implemented, as described below.

#### LAR1: Menu Redesign Based on New Healthy Offerings

In LAR1, the restaurant decided to move forward with two main changes: (1) the addition of new menu items based on a new component — a hardier version of a salad, including ingredients found in the traditional cuisine — and (2) redesigning the menu to feature pre-made bowls (including two healthier options, with the new vegetable offering) and eliminate a menu heading that highlighted fried foods, renaming it from frieds to snacks. The new vegetable component, initially called roasted, sofrito seasoned veggies (Verduras) included cabbage and a mix of other healthier vegetables (pumpkin, peppers) used traditionally in the cuisine. The dish underwent in-house testing, led by the chef. The menu redesign was done collaboratively between the research team and the restaurant owner, undergoing several rounds of revision before a quick test (soft-launch) to ensure clients and staff understood the new menu format and terminology. The process of developing and prototyping the updated menu required us to factor in both the consumers’ experience while ordering — the restaurant owner said that even though the original menu starts with the rice base, customers typically started with the type of protein they wanted in their bowls — as well as the restaurant’s computer system — similar to the original menu, orders were originally put into the computer system starting with the rice base, so the staff had to guide customers through the order process. Additionally, we factored in new customers’ unfamiliarity with the menu, introducing Signature Bowls to help guide their decision-making process. These pre-built bowls included best seller combinations and two new combinations, featuring the healthier verduras as base (instead of white rice) and as side, including a vegetarian option. The owner also agreed to eliminate a section highlighting fried snacks. While the fried offerings remained, these were placed under a Snacks section, which also allowed the restaurant more flexibility in adding new, non-fried snacks into this section of the menu in the future. The previously vegetarian option, called sin carne (without meat) was eliminated, with beans and verduras serving as additional mains for the menu. While the Sin carne was a bean-based bowl, the naming of the dish as without meat implied gave a sense of lack or deprivation, whereas listing beans as a main (alongside verduras) allowed clients to see these as equally important mains, as the animal-based offerings (chicken, pork, shrimp). Other additions included seltzer water as an alternative to plain water and sugary beverages and avocado slices as a healthy side dish traditionally consumed in the cuisine. Working with the owner and chef, the new vegetable offering (Verdura) was incorporated to different parts of the menu, moving beyond just a new side, as was the case of the green salad that was in place before.

#### LAR2: Social Media Promotion of Existing Healthier Items

In LAR2, the owner decided to move forward with a revamping of the social media strategy, with a focus on Instagram and Facebook, as the main platforms used at the time. We informed the process by seeking examples of similar restaurants showcasing healthier options in an appealing way, where these offerings were promoted primarily based on their sensorial enjoyable qualities and connections to the heritage cuisine. This approach was informed from our research, as well as other work that has documented how customers associate dishes labeled with deprivation and blandness, and not with the enjoyment and indulgence typically associated with eating out (Jun & Arendt, 2016). We worked with the owner and our team’s dietitian to identify the healthier items on the menu. These items were those identified as nutrient-rich, with known health benefits. Specific examples included items such as avocado (healthy fats), beans (high fiber content), vegetables, fish, and seafood.

The testing period involved a team member initially working with the owner to create posts and a schedule that would be feasible and promote healthier choices in the restaurant. The prototyping was focused on strategies that would ease the owner’s engagement in social media, seeking to address initial barriers identified. These included lacking appealing images for highly visual social media channels (as in the case of Instagram) and providing the owner for initial messaging and information to facilitate the image’s accompanying text. The team worked with the restaurant partners and engaged in further research to develop a toolkit of potential posts and keywords to highlight dishes’ flavor and historical connections to the cuisine. During this time, the team had ongoing discussions with the owner, allowing us to fine-tune postings and develop a toolkit for the owner to use to facilitate postings. We worked with a professional food photographer to provide the owner with a bank of new images to draw from, featuring these healthier dishes in an appealing way. The resulting intervention involved the owner continuing to devise these postings, seeking to increase the visibility of the healthier dishes, along with ongoing communication with the team to provide guidance as needed, facilitated through our ongoing monitoring of the owner’s social media activities and team check-ins.

## Discussion and Implications

In this manuscript, we set out to describe the process of applying HCD alongside the Behavior Change Wheel to devise tailored interventions in two Latin American restaurants, engaging restaurants as a vehicle for health promotion via participatory approaches that leveraged their existing needs and strengths. In this study, our integrated use of HCD and the Behavior Change Wheel guided our conceptualization of the problem and the selection of interventions and implementation strategies. We also described specifically the use of the COM-B model for behavioral diagnosis and intervention function identification, along with the APEASE criteria to select the changes to be implemented. The resulting tailored interventions corresponded to identified areas for change based on the COM-B diagnosis, as follows: In LAR1, the intervention focused on increasing the availability of healthier options, corresponding to the need to address physical opportunity through the environmental restructuring intervention function. In LAR2, the intervention focused on changing perceptions of healthier options, corresponding to the need of changing knowledge (psychological capability) and reflective motivation, COM-B drivers associated with the education intervention function.

Our application of HCD was designed to facilitate a process of learning and discovery through our research and discussion with the owners and staff, which eventually enhanced the development and implementation of changes with the potential of facilitating healthier choices in these establishments. The changes were driven by the restaurants, with our team serving as facilitators to first motivate and then fine-tune and support the desired changes. This user-centered approach was essential in our collaboration, ensuring a relationship of trust and respect that greatly enhanced the process and our understanding about the perspective and challenges faced by this sector. While the resulting changes were limited to owner’s preferences, interests, and operational capacity, our application of HCD opened the doors for change to happen in the first place, even if limited, resulting in changes in attitude about engaging in changes in business practice, particularly in LAR1. The changes implemented responded to the perceived needs of customers and the level of feasibility for restaurant owners and staff. Additional strategies that may have had a greater impact on healthier menu item sales may have been viewed as irrelevant or not of interest in the establishments. At the same time, the alternative approach of implementing researcher-driven ideas from the get-go may simply be unsustainable, infeasible, and a waste of resources. Top-down approaches may even potentially close the opportunity for restaurant owners to open the door to future collaborations with public health researchers if relationships are perceived as uneven or not truly accounting for their lived experience in the sector.

### Limitations

At the same time, the application of this approach has limitations and potential barriers that need to be taken into consideration. HCD is a new approach to developing interventions, digressing from other commonly used approaches, where, for instance, public health researchers approach community sites with an intervention or change already in mind. While our partner restaurant owners appreciated our co-development approach, this also confused in the beginning, requiring owners who were comfortable with the uncertainty and open to the process, a factor that was facilitated via the building of trusting relationships. Hence, this process requires time from all parties involved, which is a barrier when working with restaurant owners. Our team worked within their schedules, requiring a high level of flexibility. Another consideration is the need to be open about problem and solution definition when applying HCD, which requires the breaking of hierarchies among all involved, to shift views of who holds the expertise to facilitate innovations. Our HCD application was facilitated by our multidisciplinary team, including the participation of a designer with a background in food studies, who assisted in the development and facilitation of the workshops with restaurants, bringing key know-how on approaches to promote innovative thinking, with an understanding about the complexities involved in working with the sector.

The integration of HCD and the Behavior Change Wheel approaches is novel in public health, with notable exceptions particularly in the realm of technology-based interventions (Hendrie et al., 2019; Huang et al., 2020). In agreement with past conclusions derived from these studies, the use of HCD enhances the application of the Behavior Change Wheel framework, moving beyond more traditional data collection approaches to elicit user-driven innovations (Huang et al., 2020). The HCD process has empathy at its core, where the research team needs to understand and accommodate the process from the business perspective and allow the stakeholders to guide the direction of the process. This user-driven approach is a key strength of HCD, with the potential to enhance intervention design and implementation approaches, as is the case of the Behavior Change Wheel. Furthering public health engagement with HCD has the potential to move the needle on addressing diet-related health disparities by centering our focus on community needs and preferences. While our process uncovered tensions between the business and public health goals, these tensions are front and center in the process of iterating to make realistic changes in a dynamic system, in this case, restaurants, but more broadly, in a wide range of public health-focused community settings.

## Conclusion

Implementation science is yet to be fully applied to inform and examine interventions implemented in complex community settings, particularly in under-engaged sectors such as Latin American restaurants. This work expands the application of this approach, enhanced by the use of HCD, following emerging trends and calls to bridge these approaches closer together to enhance the operationalization and uptake of evidence-based public health strategies (Chen et al., 2021; Dopp et al., 2019; Gottgens & Oertelt-Prigione, 2021). While HCD shares many characteristics with community-based participatory research, HCD can enhance collaboration and resulting interventions through an explicit focus on creativity, innovation, and the inclusion of multi-sectoral and multi-disciplinary perspectives (Chen et al., 2021). This distinction has immense potential to elucidate new solutions to persisting, complex problems, meriting the need for future work and engagement with HCD methods. Hence, HCD has the potential to be an innovative way to engage restaurants by ensuring that projects seek to include and meet their needs. The work presented is an advance on previous applications of HCD in public health (Bazzano et al., 2017), applying both HCD and the Behavior Change Wheel in an innovative setting and building on the sparse literature applying HCD to healthy nutrition (individual-focused) interventions (Cradock et al., 2022; Joshi et al., 2019).
